# Supplementary figures and images for: Decoding decision-making behavior from sparse neural spiking activity
Source: PLoS Comput Biol. 2025 Aug 21;21(8):e1013335. doi: 10.1371/journal.pcbi.1013335 (PMC12416849; doi:10.1371/journal.pcbi.1013335)

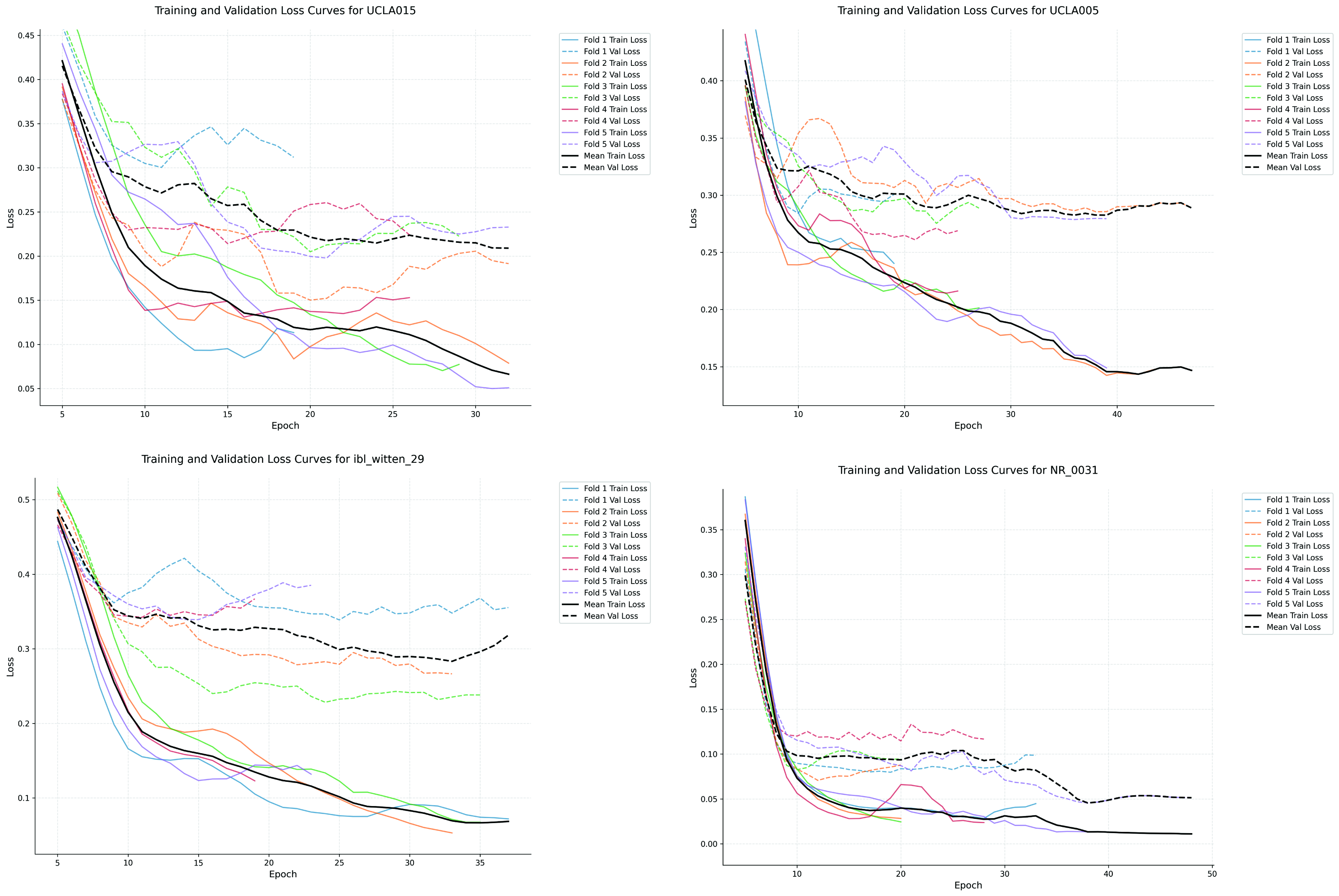

Supplement: S1 Fig — (TIF) [file pcbi.1013335.s003.tif]

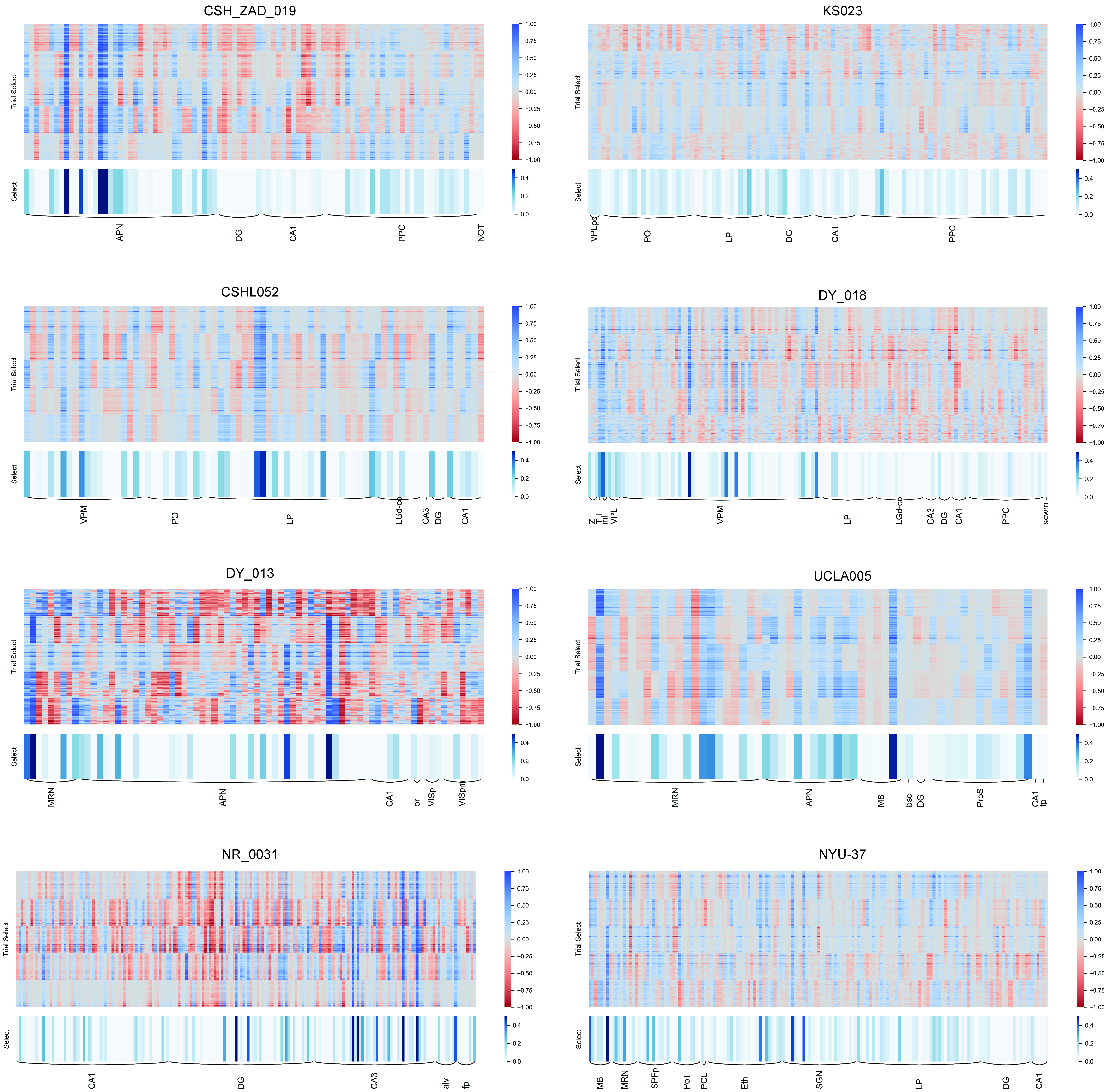

Supplement: S2 Fig — (TIF) [file pcbi.1013335.s004.tif]

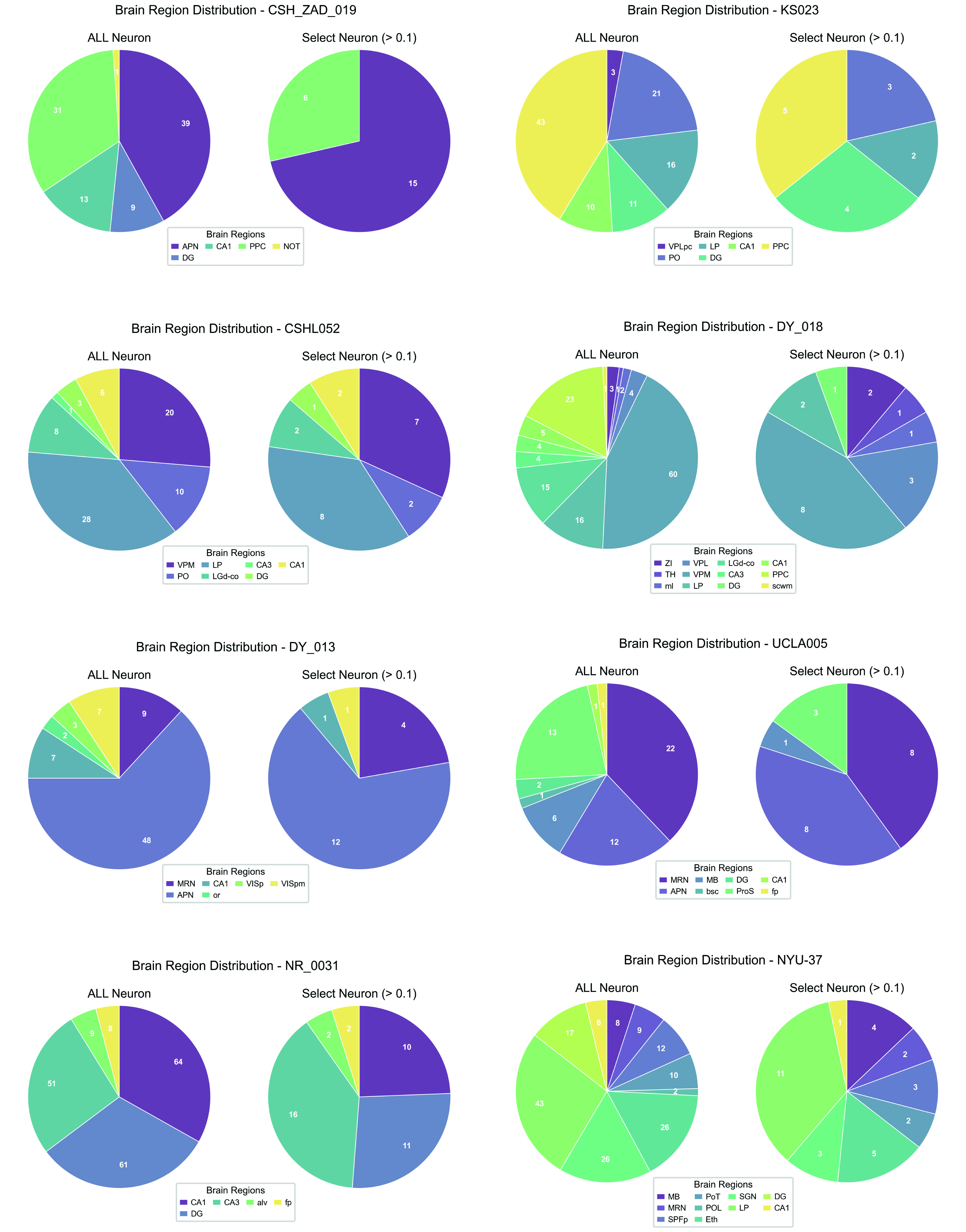

Supplement: S3 Fig — (TIF) [file pcbi.1013335.s005.tif]

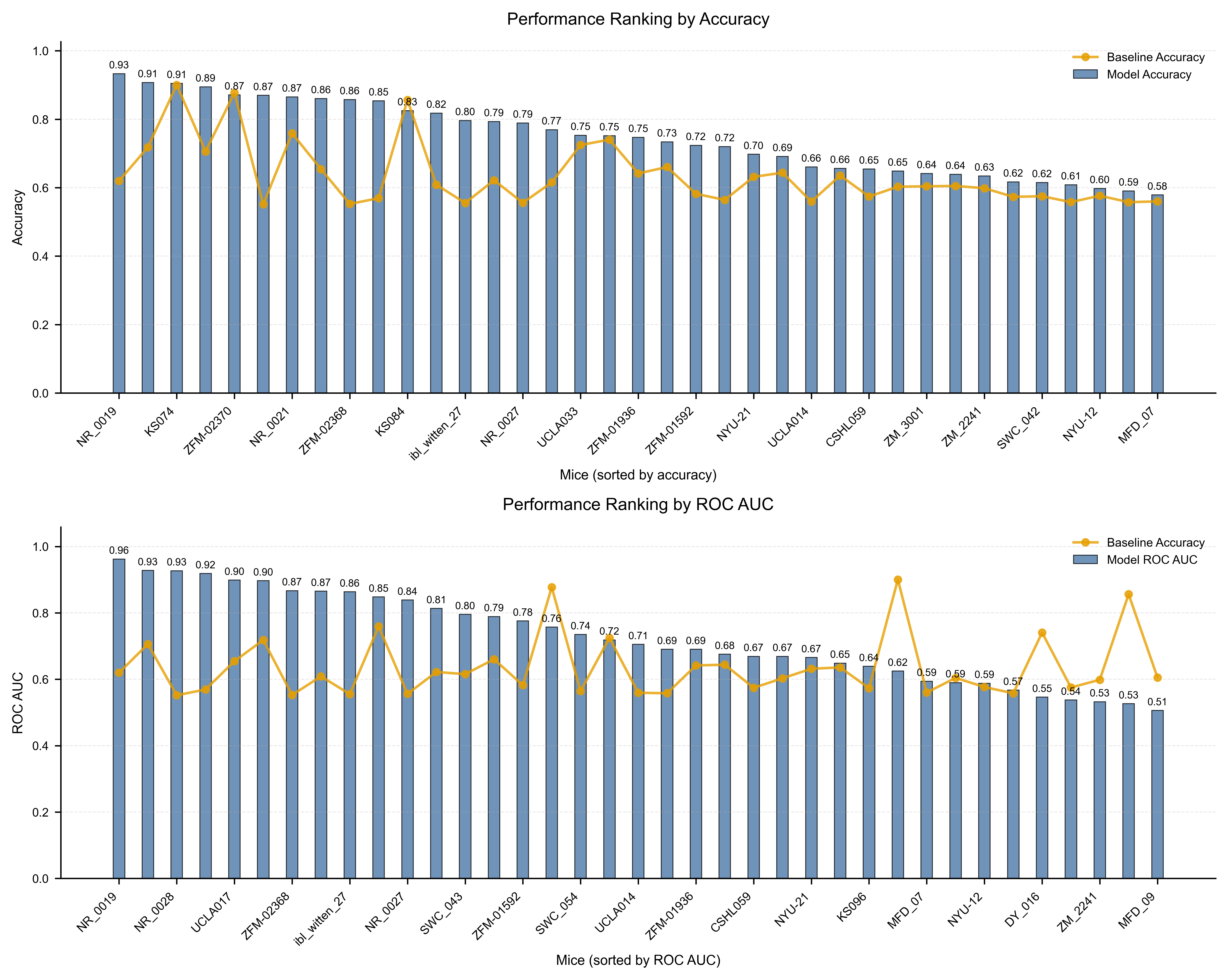

Supplement: S4 Fig — (TIF) [file pcbi.1013335.s006.tif]
