# Supplementary material for: Decoding decision-making behavior from sparse neural spiking activity
Source: PLoS Comput Biol. 2025 Aug 21;21(8):e1013335. doi: 10.1371/journal.pcbi.1013335 (PMC12416849; doi:10.1371/journal.pcbi.1013335)
Supplement: Attachment — Submitted filename: review_response.pdf [file pcbi.1013335.s007.pdf]

Responses to Reviewers' Comments for Manuscript PCOMPBIOL-D-24-01470

Decoding Decision-Making Behavior from Sparse Neural Spiking Activity

Addressed Comments for Publication to

PLOS Computational Biology

by

Yuhang Zhang, Tao Sun, Boyang Zang and Wan Sen

Dear Dr. Daniele Marinazzo,

Please find enclosed the revised version of our previous submission entitled "Decoding Decision-Making Behavior from Sparse Neural Spiking Activity" with manuscript number PCOMPBIOL-D-24-01470. We would like to thank you and the reviewers for the valuable comments which help improving the quality of our manuscript. In this revision, we have carefully addressed the reviewers' comments. A summary of main modifications and a detailed point-by-point response to the comments from Reviewers 1 and 2 (following the reviewers' order in the decision letter) are given below.

Due to updates in the data presented in the manuscript, the entire experimental section has been revised. Since highlighting the changes in the document would affect the clarity of the presentation, we have refrained from using highlights to indicate the revisions. We kindly ask the reviewers for their understanding regarding this matter.

Sincerely,

Yuhang Zhang, Tao Sun, Boyang Zang and Wan Sen

Note: To enhance the legibility of this response letter, all the editor's and reviewers' comments are typeset in boxes. Rephrased or added sentences are typeset in color. The respective parts in the manuscript are highlighted to indicate changes.

Response to Reviewer 1

Comment 1.1

Superiority Over Simpler Models is Unclear: While the attention mechanism is stated to improve localization of task-relevant neurons and regions, its advantage over simpler model selection techniques (e.g., feature selection or regularized regression models) is not convincingly demonstrated. The dynamic nature of the attention mechanism's contribution to overall model performance remains vague.

Response 1.1: Thank you for the comment.

We sincerely appreciate your insightful question regarding the comparative advantage of our attention mechanism over simpler model selection techniques. To address this concern comprehensively, we have implemented the following improvements:

We have conducted an extensive comparative analysis using multiple neuronal selection methods, including CA-BiLSTM, L1-regularized Logistic Regression (L1-LogReg), L1-regularized Multi-Layer Perceptron (L1-MLP), Analysis of Variance (ANOVA), and Recursive Feature Elimination (RFE). The efficacy of these selection approaches was systematically validated using both SVM_RBF (Figure 7a) and BiLSTM models (Figure 7b). Importantly, when evaluated across both accuracy and AUC metrics, neurons filtered by our CA-BiLSTM model consistently demonstrated superior performance compared to those selected by other methods. These results provide compelling quantitative evidence that our attention mechanism offers significant advantages in neuronal selection compared to traditional feature selection and regularized regression approaches, further validating the effectiveness of our proposed methodology.

Comment 1.2

Neglect of Fine Temporal Spike Patterns: The model's approach to partitioning spike data appears to overlook the fine-grained temporal structure of spike trains, which may encode critical decision-making information. By treating partitions coarsely, the model risks discarding temporal dynamics that could provide deeper insights into the neural code underlying behavior.

Response 1.2: Thank you for the comment.

We sincerely appreciate the reviewer's valuable feedback. We fully recognize the critical importance of temporal fine structure in understanding neural coding, and have conducted comprehensive experimental comparisons and analyses to address this concern:

We specifically designed control experiments to systematically compare traditional time-interval-based partitioning methods with our proposed segment-based approach. As shown in Figure 2b, our segmentation method demonstrates significantly higher accuracy than time-interval partitioning.

Our experimental observations reveal substantial variability in decision response times across different trial rounds in mice. Traditional time-interval alignment methods implicitly assume that mice maintain consistent neural states at identical time points—an assumption that may oversimplify the complex dynamic characteristics of the decision-making process.

Our core concept involves partitioning based on functional phases of the decision pro-

cess—specifically, we hypothesize that mice undergo the same number of thinking stages prior to each decision, though the duration of these stages varies across trials. Based on this hypothesis, we implemented a uniform time segmentation scheme for the current number of segments. It is worth noting that we also explored non-uniform time segmentation schemes and more complex partitioning strategies, but these approaches did not yield significant performance improvements. This finding suggests that our current uniform partitioning approach achieves an optimal balance between computational complexity and capturing temporal dynamic features.

In our future work, we plan to further investigate how to more effectively integrate fine-grained temporal information of neural activity while maintaining model interpretability.

Comment 1.3

Marginal Improvement Over Baselines: The improvement of the CA-BiLSTM over other methods, such as convolutional neural networks (CNNs), is marginal. This raises questions about the utility of the additional complexity introduced by the CA-BiLSTM framework.

Response 1.3: Thank you for the comment.

We appreciate the reviewer's observation regarding the performance comparison between our CA-BiLSTM and baseline methods. We would like to address this concern with the following clarifications:

The CA-BiLSTM model effectively identifies decision-relevant neurons while leveraging the inherent temporal processing capabilities of BiLSTM architecture, which is particularly well-suited for analyzing decision-making time sequences in mice. This alignment between model architecture and the sequential nature of neural decision processes provides interpretability advantages beyond pure performance metrics.

Our comparative CNN models, despite showing competitive performance, lack the ability to effectively extract important neurons from the neural population. It's worth noting that the CNN baseline results presented are not from standard implementations, but rather from carefully optimized multi-channel convolutional architectures with extensive hyper-parameter tuning to maximize their effectiveness. The CA-BiLSTM achieves superior interpretability while maintaining competitive performance without requiring the same degree of architecture-specific optimization.

We believe these considerations justify the additional complexity of the CA-BiLSTM framework, as it provides both competitive performance and enhanced interpretability for understanding neural decision processes.

Comment 1.4

Handling of Spike Train Sparseness: The study highlights the sparseness of spike trains as a challenge in its introduction and title but fails to clearly articulate how this issue is addressed. The partitioning of data, as described, appears equivalent to coarse binning.

Response 1.4: Thank you for the comment.

We sincerely appreciate your thorough review and valuable feedback on our paper. A fundamental challenge in decoding decisions from neural data is the inherent sparseness

of spike trains, where individual neurons fire infrequently and irregularly. Our approach addresses this challenge through several strategic mechanisms:

Adaptive Temporal Segmentation: Unlike fixed time bins that may exacerbate sparsity issues, our equi-segment preprocessing adaptively scales to the reaction time of each trial. This approach ensures meaningful temporal patterns are captured while minimizing the number of empty bins that would result from finer temporal discretization. Additionally, since reaction times vary significantly during the decision-making process, traditional fixed time interval methods lead to inconsistent data dimensionality across different trials. Our equi-segment method uniformly divides the decision time of each trial, ensuring standardized representation of data across trials—a critical factor for effective processing of sparse neural signals.

Channel Attention Mechanism: The attention component of our CA-BiLSTM specifically addresses the sparsity issue by dynamically weighting neuronal contributions. This approach allows the model to focus on information-rich neurons while reducing the influence of uninformative or silent neurons, effectively improving the signal-to-noise ratio in sparse datasets.

As demonstrated in the comparative experiments shown in Figure 2, this approach significantly enhances prediction accuracy across all mouse samples compared to fixed time window binning.

Comment 1.5

Limited Insight into Decision-Making: While the attention mechanism highlights important neurons and brain regions, it remains unclear what new insights this approach provides into decision-making processes. The conclusion that some neurons or regions are more important than others is already well-established in neuroscience.

Response 1.5: Thank you for the comment.

We sincerely appreciate the reviewer's valuable feedback.

Our paper primarily offers a more refined methodology for the selection and identification of decision-related neurons within specific brain regions, rather than claiming fundamentally new neuroscientific discoveries. The established neuroscientific understanding that certain neurons or regions contribute more significantly to decision-making processes actually serves to validate the effectiveness of our approach.

The primary contribution of our work lies in the methodological advancement that enables more precise and automated identification of these critical neurons during dynamic decision processes. This computational approach complements existing neuroscientific knowledge by providing a data-driven, quantitative framework for neuron selection that can be applied consistently across different experimental paradigms and datasets.

While building upon established neuroscientific principles, our method offers enhanced analytical precision and interpretability that could facilitate future research requiring fine-grained temporal analysis of decision-making circuits.

Response to Reviewer 2

Comment 2.1

Structural comments
Before pointing out specific comments about the scientific content of the manuscript, I feel obliged to mention a few high level comments regarding its structure:
- The discussion (or conclusions, as the authors write) is very short with just 2 paragraphs, and the first one being a summary of the findings. I think there are many important nuances (as I will get into later) that could have been discussed, but the second paragraph just discusses things without much substance about the paper.
- The supporting information is very lacking. There was only one supplementary figure that I could find, with an *incorrect* caption, which is a copy of Figure 3's (but the figure itself is more like Figure 5 for each mice). There are many details that could have been provided, and which also I will elaborate on further.
From the get-go, these two structural concerns undermine my confidence on the quality of the work that is being presented.

Response 2.1: Thank you for the comment.

We sincerely appreciate the reviewer's constructive feedback regarding the structural aspects of our manuscript. We have addressed these concerns thoroughly:

We have completely revised the discussion section to provide a more comprehensive analysis of our findings and their implications. The expanded discussion now explores the nuances of our results, contextualizes our work within the broader field, addresses limitations, and outlines more concrete directions for future research.

Regarding the supporting information, we acknowledge the deficiencies in our original submission. We have now substantially enhanced the supplementary materials with:

1. Corrected captions for all supplementary figures
2. Additional figures providing detailed results for individual mice
3. Comprehensive methodological details that support the reproducibility of our work
4. Technical specifications of model architecture and hyperparameter configurations

We have carefully reviewed all figure captions throughout the manuscript and supplementary materials to ensure accuracy and clarity. The supplementary materials now provide a robust foundation for the claims presented in the main text.

We appreciate the reviewer highlighting these structural concerns, which have allowed us to significantly improve the quality and rigor of our presentation. We believe these revisions address the reviewer's concerns and strengthen the overall scientific contribution of our work.

Comment 2.2

Model architecture
I would have liked to see some more justification to motivate the architecture of the attention module. In particular, the outputs of the AvgPool and MaxPool operation is fed to an MLP of a certain size. I couldn't even find the details of the size chosen, but why this one? How was this hyperparameter selection done? Also, the pooling operations are indicated to be done 'along the time dimension'. Does this include both time along the trial, and trial number? The output E_c seems to be of size 1xN, where N is the number of neurons (is it? N is not properly defined). I can't find information about the trial dimension. How is the data batched? All this is crucial to then understand the implications of the selection on neurons based on attention weights later on. This part needs to be very clear, and for me at least, at the moment it isn't.

Response 2.2: Thank you for the comment.

We appreciate the reviewer's detailed questions regarding our model architecture. We have expanded the description to provide greater clarity:

The MLP in our attention module uses an intermediate feature dimension of 128. This is indeed a hyperparameter that can be adjusted. After extensive hyperparameter tuning experiments, we found this value provided an optimal balance between expressive capacity and computational efficiency across our datasets.

Regarding data dimensions, our input has the shape (trials, segments, neurons), where: - Trials varies by mouse (specific values are provided in Table 1) - Segments represents our temporal division of each trial - Neurons corresponds to the number of recorded neurons (N)

For clarity on the pooling operations:

Temporal dimension pooling: - This specifically refers to pooling along the time steps (T) dimension - Purpose: To capture temporal features across the entire time sequence, such as activity patterns of a neuron across all time points - Input: [Batch, TimeSteps, Channels] $\rightarrow$ Output: [Batch, 1, Channels]

Spatial dimension (channel) pooling: - "Spatial" here refers to the neuron dimension (C) - Purpose: To capture spatial relationships between different neurons, such as the activation state of all neurons at a particular moment - Input: [Batch, TimeSteps, Channels] $\rightarrow$ Output: [Batch, TimeSteps, 1]

The temporal dimension pooling captures long-term dependencies and neural temporal activity patterns, while spatial dimension pooling captures relationships between different brain regions and spatial distribution features.

As the reviewer correctly noted, Ec has dimensions $1 \times N$, where N is the number of neurons. The trial dimension varies by mouse, with the specific values detailed in Table 1 of our manuscript.

We have included a comprehensive table in the revised manuscript that further explains our parameter selection process and architectural choices to address these important details.

Comment 2.3

Data processing
Going now first into trial selection, the 'screening criteria' imposed seem to be quite strict. In the end only 13 sessions of 13 mice survive the process, out of 82 initial mice recording sessions, as per the IBL paper following their exclusion criteria (121 originally). I think it is important that the authors indicate the percentage of sessions that gets discarded in each step, and if it's high they justify the reasons for the thresholds they considered. For instance, how sensitive were the results to the threshold for the minimum number of trials per session? How about the bias criterion? Perhaps using AUC (Area under the ROC curve) instead of classification accuracy to quantify the performance of the model would have allowed to be laxer in terms of class imbalance. Or the data could have been subsampled to balance the classes.
Then, regarding the pre-processing and binning of spike times, I think this is an important step that raises some questions. The choice of the 'segmentation method' is surprising, given the disparity of reaction times (RTs) in the data. The authors show a high correlation between the performance of the chosen segmentation and that of traditional fix window binning of 30ms, with the former slightly outperforming the later. Was the 30ms size the best possible one? It so, it should be stated. The segmentation method leaves me with some doubts, because it assumes some implicit temporal scaling of neural activity, but I don't see why this would be the case. A good sanity check would be to show that, given this segmentation, the model doesn't perform much worse on trials with long reaction times compared with short reaction time ones, which are the predominant type. The whole speed-accuracy tradeoff in the task seems challenging to be reproduced with this selection.

Response 2.3: Thank you for the comment.

We appreciate the reviewer's insightful questions regarding our trial selection criteria and data processing methodology.

As the reviewer correctly noted, the International Brain Laboratory (IBL) dataset has expanded since our initial submission. While we originally used data from 42 mice, IBL has subsequently released additional data bringing the total to 104 mice.

Regarding our screening criteria, the IBL standard recommends a minimum of 400 trials per session, while we set our threshold at 250 trials. This reduction was carefully considered to balance dataset size with training quality. If the minimum trial count is too small, there are insufficient samples for effective training and learning. We have added a comprehensive breakdown in the revised manuscript showing the percentage of sessions filtered at each step of our selection process.

In response to the reviewer's suggestion about evaluation metrics, we have now incorporated Area Under the ROC Curve (AUC) alongside accuracy to quantify model performance. These dual metrics provide a more comprehensive assessment of model efficacy, particularly in scenarios with class imbalance (Figure 4 in the revised manuscript).

For the temporal binning question, we systematically tested fixed window sizes ranging from 10ms to 50ms and found that 30ms yielded optimal performance for the CA-BiLSTM model. We have included these comparative results in Table S1 of the supplementary materials.

Following the reviewer's excellent suggestion regarding reaction time effects, we analyzed

the differences between correctly and incorrectly predicted trials in terms of reaction time and stimulus strength. Interestingly, we found that correctly predicted trials had slightly longer mouse reaction times compared to incorrectly predicted trials, with an average difference of 11 milliseconds (t-test p-value = 0.031). This suggests a speed-accuracy tradeoff that aligns with established neuroscientific principles.

Regarding class imbalance, we tested resampling techniques on mice with highly imbalanced classes. However, performance remained suboptimal, likely because the total number of experimental trials for these mice was too small for the model to effectively capture decision-making information, even with resampling adjustments.

Comment 2.4

Task
The IBL task is by now a well stablished experimental paradigm, and it has some special features that are important, but that the authors did not mention. In particular, after the first 90 trials, the trials follow a blocked fashion in which the probability of presentation of the stimulus in one side is higher than the other. There are also different stimuli contrasts, and additional, variability in the RT from trial to trial. Ideally, all of these features could have been taken into account to test in more detail the robustness and performance of the model.
Particularly, rather than just focus on the choice prediction accuracy, it would be interesting to also see what happens when the model misclassifies a trial. Do those classification errors correspond predominantly to difficult trials, or to trials with a particular RT range? Additionally, does the model make the same type of mistakes as the animals, given the stimulus? This would add deeper insight into the value of the model besides a single, global accuracy score that is a few percent higher than that of, for instance, a MLP.

Response 2.4: Thank you for the comment.

We sincerely appreciate the reviewer's valuable insights regarding the IBL task's specific features and the suggestion for deeper analysis of model performance.

Following your recommendations, we have conducted a more comprehensive analysis comparing trials where our model made correct predictions versus incorrect predictions. This analysis reveals several interesting behavioral and neural correlates of model performance:

As shown in Figure 7c of our revised manuscript, we found meaningful differences between correctly and incorrectly predicted trials in terms of both reaction time and stimulus contrast. Intriguingly, trials correctly predicted by our model exhibited slightly longer mouse reaction times compared to incorrectly predicted trials, with an average difference of 0.015 seconds (t-test p-value = 0.031). Additionally, the reaction time distribution for correctly predicted trials was more tightly clustered, suggesting more consistent neural patterns in these trials.

Importantly, we found that correctly predicted trials had significantly higher screen stimulus contrast than incorrectly predicted trials. This finding aligns perfectly with expected behavioral outcomes, as higher contrast stimuli typically enable more accurate judgments by mice. This suggests our model struggles with the same difficult trials that challenge the animals themselves.

We have also incorporated analysis of the blocked trial structure (where stimulus presentation probability is biased toward one side after the first 90 trials) in our revised manuscript.

This analysis reveals how our model adapts to changing probabilistic contexts, providing further evidence of its ability to capture meaningful decision-related neural dynamics.

These additional analyses demonstrate that our model's performance advantage over simpler approaches like MLPs is not merely reflected in slightly higher accuracy, but in its ability to capture behaviorally relevant patterns in the neural data that align with established principles of decision-making processes.

Comment 2.5

Attention mechanism
The last third of the manuscript is employed to assess the worth of the proposed attention mechanism in order to determine the impact of different neurons to the mice decisions. The strategy employed consists on ranking the neurons by their attention weight or score, and then training a *different* model architecture without an attention mechanism, in order to show that using the neurons with higher attention weight improves the classification performance as opposed to the same number of randomly selected neurons, or all the neurons. I see this as a valid sanity check, but it is hardly surprising. If the attention model is trained successfully, how could it have been otherwise? Furthermore, I don't understand why the test was performed on a support vector machine, instead of the same BiLSTM architecture just without the attention mechanism. No justification whatsoever is given for this.
The attention mechanism is not the only way to perform feature extraction in ML models. The authors could consider comparing it to simpler methods. For instance, it could be compared with an MLP with L1 regularization in the input weights, which would be a form of context-independent feature selection. After all, the crucial aspect of the attention mechanism is that it's dynamic and adaptive. Hence, the dynamic aspect should be tested and contrasted with a non-dynamic method. Another strategy could be to leverage the task blocks, and to train the model in only one of the block types and test in the other, repeating then the procedure with the opposite type. How does the performance degrade? Are there block specific neurons that are sensitive to the bias? Finally, the attention mechanism could be validated with ground-truth simulated data, perhaps using a Poisson-GLM where one can choose the particular weights of each neuron towards the choice.
Finally, something surprisingly lacking is the combined assessment of the distribution of "important" neurons across brain areas and mice. Are these neurons predominantly located in areas previously identified as decision relevant? All that is shown is distributions for an example mouse from the Witten lab, that doesn't particularly conform to the expectations of distribution of important areas that was primed before as decision relevant, such as LP and PPC (this animal has only 2 PPC neurons, and none of them are selected by the attention mechanism). I believe showing this distribution across mice is crucial to further validate the proposed attention mechanism and position it as a competitive tool to retrospectively identify task relevant neurons.

Response 2.5: Thank you for the comment.

We greatly appreciate the reviewer's insightful feedback regarding our attention mechanism evaluation. Following these suggestions, we have conducted a more comprehensive analysis as shown in Figure 7 of our revised manuscript.

To address the concern about comparing our attention mechanism with alternative feature selection methods, we have implemented a thorough comparative analysis. Figure 7a,b presents validation results across multiple neuron selection methods including CA-BiLSTM, L1-LogReg, L1-MLP, ANOVA, and RFE. We evaluated these selections using both SVM_RBF (Figure 7a) and BiLSTM models without attention (Figure 7b) as validation architectures. Across both accuracy and AUC metrics, neurons selected by our CA-BiLSTM model consistently outperformed other selection methods, providing stronger evidence for the effectiveness of our attention mechanism beyond simple feature selection.

The comparison with L1-regularized models (both logistic regression and MLP) directly addresses the reviewer's suggestion to compare with context-independent feature selection methods. Our results demonstrate that the dynamic, context-sensitive attention mechanism provides meaningful advantages over these static approaches.

Regarding the distribution of "important" neurons across brain regions, we have conducted a comprehensive analysis across all mice in the dataset. Within the five key brain regions focused on in the IBL dataset, we found that the DG, LP, and PPC regions consistently showed significant importance in the decision-making process, aligning with existing research findings (refs [31,32]). This cross-mouse analysis provides stronger validation for our attention mechanism as a tool for identifying task-relevant neurons.

Interestingly, we also observed that neurons in the anterior tegmental nucleus, suprachiasmatic nucleus, and ventral posterolateral thalamic nucleus showed substantial contributions to visual decision prediction in some mouse samples. However, since these regions were not primary targets of IBL recordings and were only detected in a limited number of mice, these findings, despite their high average attention scores, may contain some randomness due to the limited sample size and warrant further investigation in future studies.

This comprehensive cross-region, cross-mouse analysis significantly strengthens the validation of our attention mechanism and positions it as a valuable tool for retrospectively identifying task-relevant neurons in complex neural datasets.

Comment 2.6

Detailed comments
Now, I will refer to more specific comments regarding details in the text. - Consider replacing words such as "linchpin" and "winnowing" by simpler words (lines 11 and 51). - Line 60: "preceding decision-making tasks" do you mean "preceding decisions"? - Lines 61, 78: decision making "direction" - the task hasn't been introduced yet, so "direction" should be avoided here because it's not obvious what it means, rather use "action". - Lines 72-76: I don't fully get why the "pulse data" is emphasized here, since the spikes are eventually binned. It's not like the raw ephys signal is taken as input. - Lines 81-83: This is largely repeated, was said already in lines 68-70. - Line 97: "maintain a time-step memory at time step t". This is confusing, do you mean "a one-time-step memory"? - Line 119: Consider rewording, performing inference or evidence accumulation is not equivalent to being "constantly contemplating their decision-making" - Line 121: I disagree with the choice of words "instinctively" and "conscious". For the first, I would rather use "impusively", and the second incorrectly suggests a conscious experience during evidence accumulation. - Line 122: This needs clarification. - Line 136: "the form of the algorithm" replace by "algorithmic form". - Task description: there is no mention of the block structure of the task, the fact that there are different contrasts, or that the task is a reaction time task such that the animals control the duration of the stimulus. - Table 1: Number of good "nuerons". - Line 199: Avoid repeating "decision-making times". - Line 222: "resulting some loss of temporal information" - given that the spike counts are binned in a few segments, is it really the case? - Fig. 3: "under different segmentation" - missing schemes? - Line 242: How was the significance assessed? You must report this. - Fig. 4: Missing y label in a). - Fig 5: The caption in this figure needs to be clarified. What is the score value? Also it says "nueron" ibl_witten_29 - Line 264: Missing figure? - Line 266: "consistent with the current research findings" - do you mean your findings? This hasn't been shown. - Line 282: Again, why the SVM and not a biLSTM? - Line 290: What exactly is the selection threshold apply onto? This has not been explained. - Fig. 6: brain "eegion". Also filtering threshold of "0. 1" (extra space). Also the caption should indicate which of the pie charts corresponds to what. - Fig 7: this is the first time that it's said what "SVM_RBF" means.

Response 2.6: Thank you for the comment.

We sincerely appreciate the reviewer's thorough examination of our manuscript. We have addressed each point as follows:

We have replaced "linchpin" with "key" and "winnowing" with "selecting" to improve clarity.

We have corrected the phrase to "preceding decisions" as suggested.

We have replaced "direction" with "action" throughout the text when referring to decision outcomes before the task introduction.

We agree with the reviewer's observation. We have modified our description from "Neuronal action potentials manifest as sparse temporal sequences of pulse-like data" to "Neural activity is encoded as temporal sequences of binned spike counts" to more accurately reflect our methodology.

We have eliminated the repetitive content in lines 72-76 and 81-83.

We have revised the confusing phrase "maintain a time-step memory at time step t" to

"maintains internal state memory at time step t" to more precisely describe how LSTM units maintain internal state information at each discrete time point in the binned spike sequence.

We have replaced "constantly contemplating their decision-making" with "evidence accumulation" and "neural evidence accumulation process" to more accurately describe the essential nature of the decision process.

We have replaced "instinctively" with "impulsive," removed references to "conscious deliberation" to avoid inappropriate assumptions about conscious experience, and adopted "evidence integration" to describe the decision process in line with objective neuroscientific terminology.

We have revised the text to: "While reaction times have been controlled during the experiment selection process, the neural evidence accumulation process may not be consistently engaged across all trials. In some cases, responses might be impulsive rather than based on extended evidence integration."

We have replaced "the form of the algorithm" with "algorithmic form" as suggested.

We have added a comprehensive description of the task structure: "In each session, during the first 90 trials, stimuli appeared with equal probability (5:5) on the left and right sides. In subsequent trials, this probability alternated between 2:8 and 8:2 ratios. Additionally, stimulus contrast varied between 100

We have corrected the typographical error in Table 1.

We have reorganized the entire paragraph to improve flow and clarity.

We have clarified that our segmentation approach preserves temporal structure while acknowledging potential limitations in fully capturing the sequential dynamics of mouse decision processes.

We have revised the description of Figure 3a to: "Figure 3a shows a comparison of two different preprocessing methods for neural spike signals. The upper panel presents the time-interval-based preprocessing method, which quantifies by counting neural spike signals within the same time interval; the lower panel presents the equal-segment preprocessing method, which uniformly divides the decision time of different trials and calculates the number of neural spike signals within each segment."

We have thoroughly revised this section for clarity.

We have added appropriate y-axis labels to Figure 4.

We have revised and supplemented the figure captions to clearly explain the "score value" and corrected the typo "nueron."

We have added Figure 7d showing the average attention scores across different brain regions for all mice: "In the five key brain regions focused on in the IBL dataset, DG, LP, and PPC regions consistently showed significant importance in the decision-making process, aligning with existing research findings [31,32]."

We have added validation experiments using BiLSTM for neuron selection.

We have clarified the selection threshold process: "The selection threshold is used to determine which neurons to retain or eliminate, with neurons having attention scores above the threshold being retained, and those below being eliminated."

We have corrected the caption to: "Comparison of brain region distribution between all neurons and attention-filtered neurons for mouse ibl_witten_29, demonstrating that the decision process is driven by a specific subset of neurons."

We have provided a full explanation: "Support Vector Machine with Radial Basis Function

Kernel (SVM_RBF)" where the term first appears.

We appreciate the reviewer's meticulous attention to detail, which has significantly improved the clarity and precision of our manuscript.

Comment 2.7

Supplementary information
The SI should include the following: - Further details about the model architectures, including the different models shown in Fig. 4. - Hyper parameter selection. - Training and validation loss examples. - Hardware specifications, and model training and evaluation times. - Details on the data selection criteria, with numbers of trials and sessions excluded at each step. - A figure with panels like Fig 2b for different bin widths for the the time interval partition, or at least a quantification of the overall performance as a function of bin width. - A figure like Fig. 6 for each mouse.
References: In general there aren't many references. At least more should be provided when mentioning earlier research on neural correlates of decision making (line 24) and the DDM (line 25). There is not citation for BiLSTMs, or attention mechanisms until late in the text (line 249).
Error bars: in general, the meaning of the error bars in the figures is not specified (figs. 3, 5, 7 and 8).
Presentation: Figures 2 and 3 could be merged. Same goes for figures 7 and 8, or even 6, 7 and 8.

Response 2.7: Thank you for the comment.

We sincerely appreciate the reviewer's comprehensive suggestions regarding our supplementary information. We have addressed all points as follows:

We have added detailed descriptions of all model architectures used in our study, including all models presented in Figure 4. These specifications are now provided in Table 3 of the supplementary materials.

We have included examples of training and validation loss curves for selected mice in Supplementary Figure S1, illustrating the learning dynamics and convergence behavior of our models.

We have added hardware specifications and training time information: "All experiments were conducted using an Intel(R) Core(TM) i5-7300HQ CPU @ 2.50GHz with a GTX1050 GPU. The average training time for five-fold cross-validation per mouse was approximately 13 seconds."

We have supplemented the main text with comprehensive data selection criteria and statistics showing the number of trials and sessions excluded at each processing step, as illustrated in Figure 2 and Table 1.

We have added a quantitative analysis of model performance across different time interval widths (20ms-50ms) showing both average accuracy and AUC metrics for all mice, which allows direct comparison with our segmentation approach.

We have included brain region distribution pie charts (before and after attention filtering) for multiple representative mice in Supplementary Figures S2 and S3. Due to space limitations, we've included a subset of mice in the supplementary materials, but the complete set of visualization results for all mice is available at our GitHub repository: https://github.com/ZhangYH321/CA-BiLSTM

We have significantly expanded our references section, adding relevant citations for neural correlates of decision-making, drift-diffusion models, BiLSTMs, and attention mechanisms throughout the text where these concepts are introduced.

We have clarified that all error bars in our figures (Figs. 3, 5, 7, and 8) represent standard deviation, providing a consistent interpretation of variability across our results.

Following the reviewer's suggestion, we have reorganized our figures by merging Figures 2 and 3, as well as Figures 6, 7, and 8, which has improved the overall flow and presentation of our results.

These additions to the supplementary information significantly enhance the transparency, reproducibility, and comprehensiveness of our study. We believe these changes address all the concerns raised by the reviewer regarding the supplementary materials.
