# Supplementary material for: Decoding decision-making behavior from sparse neural spiking activity
Source: PLoS Comput Biol. 2025 Aug 21;21(8):e1013335. doi: 10.1371/journal.pcbi.1013335 (PMC12416849; doi:10.1371/journal.pcbi.1013335)
Supplement: Attachment — Submitted filename: review_response1.pdf [file pcbi.1013335.s008.pdf]

Responses to Reviewers' Comments for Manuscript
PCOMPBIOL-D-24-01470R1

Decoding Decision-Making Behavior from Sparse Neural Spiking Activity

Addressed Comments for Publication to

PLOS Computational Biology

by

Yuhang Zhang, Tao Sun, Boyang Zang and Wan Sen

Dear Dr. Daniele Marinazzo,

Please find enclosed the revised version of our previous submission entitled "Decoding Decision-Making Behavior from Sparse Neural Spiking Activity" with manuscript number PCOMPBIOL-D-24-01470R1. We would like to thank you and the reviewers for the valuable comments which help improving the quality of our manuscript. In this revision, we have carefully addressed the reviewers' comments. A summary of main modifications and a detailed point-by-point response to the comments from Reviewers 1 and 2 (following the reviewers' order in the decision letter) are given below.

Sincerely,

Yuhang Zhang, Tao Sun, Boyang Zang and Wan Sen

Note: To enhance the legibility of this response letter, all the editor's and reviewers' comments are typeset in boxes. Rephrased or added sentences are typeset in color. The respective parts in the manuscript are highlighted to indicate changes.

Response to the Meta Review

Summary Comment 1

This revised version still leaves a lot of space for improvement, in particular some important issues were evaded or addressed unsatisfactorily. In order not to waste your time, and the time of the reviewers, I prefer to give you another chance to carefully and thoughtfully go over these points. Of course, if there's something you don't agree with, you can explain why, and stick to your version, after a proper discussion and rebuttal.
Journal Requirements:
1) We have noticed that you have a list of Supporting Information legends for S1 Table and S2 Table in your manuscript. However, there are no corresponding files uploaded to the submission. Please upload them as separate files with the item type 'Supporting Information'.
2) We have noticed that you have uploaded Supporting Information files, but you have not included a complete list of legends. Please add a full list of legends for your Supporting Information files (Support information.7z) after the references list.
3) We notice that your supplementary figures are uploaded with the file type 'Figure'. Please amend the file type to 'Supporting Information'. Please ensure that each Supporting Information file has a legend listed in the manuscript after the references list.
4) Please enter the affiliation of Yuhang Zhang in the online submission form.

Response S1: Thank you for the comment.

Thank you for your feedback. I will address the issues raised as follows:

I will upload the S1 Table and S2 Table files as separate 'Supporting Information' items. A complete list of legends for the Supporting Information files will be added after the references list in the manuscript.I will amend the file type of supplementary figures to 'Supporting Information' and ensure each file has a corresponding legend listed after the references.The affiliation of Yuhang Zhang will be entered in the online submission form.

Please let me know if further clarification is needed.

Response to Reviewer 1

Comment 1.1

Authors have addressed my questions

Response 1.1: Thank you for the comment.

We appreciate the reviewer's feedback and acknowledgment of our responses. We are pleased that we were able to successfully address all the questions previously raised. We will continue to refine our research work and look forward to future collaboration and communication.

Response to Reviewer 2

Comment 2.1

Despite the discussion having increased in length, I still find that it could have been elaborated more. There aren't any references or attempts to make meaningful connections with previous literature. There could also have been a more critical stance on the limitations of the work. The additional supplementary material is appreciated.

Response 2.1: Thank you for the comment.

We thank the reviewer for their constructive feedback regarding the need for deeper elaboration, stronger literature connections, and more critical assessment of limitations. We have comprehensively revised our discussion section to address these concerns.

We have significantly enhanced literature integration by establishing meaningful connections with seminal studies in perceptual decision-making, attention mechanisms, and sparse coding principles, properly contextualizing our findings within the broader neural decoding research framework.

We have adopted a substantially more critical stance toward our work's limitations, examining five key areas: methodological constraints (lack of cross-animal generalization testing), temporal assumptions (static vs. dynamic attention), task complexity (binary vs. multi-alternative decisions), causal inference limitations (correlational vs. causal relationships), and recording constraints (incomplete population sampling). We have also discussed potential improvements including dynamic attention mechanisms and hierarchical models for cross-animal generalization.

The revised discussion now includes deeper exploration of implications for computational neuroscience and clinical applications, providing a more balanced and comprehensive assessment of both our contributions and limitations within the systems neuroscience landscape.

Comment 2.2

Model architecture
I would have liked to see some more justification to motivate the architecture of the attention module. In particular, the outputs of the AvgPool and MaxPool operation is fed to an MLP of a certain size. I couldn't even find the details of the size chosen, but why this one? How was this hyperparameter selection done? Also, the pooling operations are indicated to be done 'along the time dimension'. Does this include both time along the trial, and trial number? The output E_c seems to be of size 1xN, where N is the number of neurons (is it? N is not properly defined). I can't find information about the trial dimension. How is the data batched? All this is crucial to then understand the implications of the selection on neurons based on attention weights later on. This part needs to be very clear, and for me at least, at the moment it isn't.
The provided additional details helps to clarify most of my doubts. I would still clarify somewhere in the text how the batching is done.

Response 2.2: Thank you for the comment.

We thank the reviewer for their continued feedback and for highlighting the need to clarify

the data batching procedure in our study. We have now added detailed information about how data batching was implemented in the "Parameter settings" subsection of the Methods section.

Specifically, we have clarified that:

1. Neural activity data was organized with each sample representing spike counts from N neurons across T time segments within a single trial.

2. Data was batched with a mini-batch size of 128 trials, resulting in input tensors of shape [128, T, N] for each training iteration.

3. T=7 represents the number of equal time segments used in our primary analysis, while N varies depending on the number of recorded neurons for each mouse.

4. This batching approach preserves temporal dependencies within each trial while enabling efficient parallel processing across multiple trials.

5. Trials were randomly sampled within each batch to prevent any potential order-related biases.

We believe these additions provide the requested clarity about how data was batched for model training, which was a concern raised in the reviewer's previous comments. This information is crucial for understanding the model's architecture and how neural information is processed across both the temporal dimension and multiple trials.

Comment 2.3

I appreciate the additional information regarding the statistics of the data selection process. It still leaves me somewhat uncomfortable the fact that so many sessions (37) are discarded due to choice imbalance, but on the other hand the authors might argue that they aimed to study close to optimal conditions as a testbed for their method. Thank you for including the AUC as an additional metric. Regarding the binning of spike times, Figure 4b raises some doubts. The accuracy curves across almost all methods don't improve as a function of the number of bins, and some of them decrease. It seems hard to justify the choice of 7 bins as optimal. This also introduces the concern that if the results are so robust to the number of bins, perhaps the temporal dynamics of the neural data are not exploited much after all by the methods. This should be clarified. I still think the implications of the scaling bins at the level of the neural code could have been discussed more. Regarding the analysis on the RTs, I'm not sure if the authors draw the right conclusions from them. They mention "Interestingly, we found that correctly predicted trials had slightly longer mouse reaction times compared to incorrectly predicted trials, with an average difference of 11 milliseconds (t-test p-value = 0.031). This suggests a speed-accuracy tradeoff (SAT) that aligns with established neuroscientific principles." While it's true in general there a SAT to be expected, when comparing correct and error trials, the stimulus difficulty should be controlled for. When this is done, there isn't necessarily an expectation for whether errors should be faster than correct responses. On the other hand, the authors also show that correctly predicted trials are associated with easier stimuli (which makes sense), and the fact that they are at the same time slightly slower contradicts the usual SAT (easier trials are faster and more accurate). Overall there seems to be confusion regarding the role of RT in the results.

Response 2.3: Thank you for the comment.

Thank you for the reviewer's valuable feedback. Your concern regarding the exclusion of 37 imbalanced sessions during the data selection process is very reasonable. We understand that this approach may give the impression of "creating ideal testing conditions for the method," but in reality, this decision was based on considerations of data characteristics and experimental validity.

Our study involves a binary classification problem, where neural spike data is inherently sparse with limited sample sizes. Under these circumstances, severely imbalanced data would lead to serious classification bias: the model tends to simply predict the majority class, which can achieve seemingly good loss function values and accuracy rates, but actually fails to effectively learn neural representation patterns related to decision-making.

To address the data imbalance problem, we attempted multiple technical approaches:

1. Introducing L1 regularization (coefficient 0.001) and L2 regularization (coefficient 0.01) to prevent overfitting

2. Enhancing minority class learning effectiveness by calculating class weights and applying Focal Loss ($\alpha = 0.25$, $\gamma = 2.0$) or weighted GCE Loss (q=0.7)

3. Using weighted random sampling techniques to increase the sampling probability of minority class samples in training batches

As shown in supplementary Figure S4, these methods still performed poorly when handling severely class-imbalanced data, with most imbalanced samples showing poor classification performance. To ensure the reliability and validity of experimental results, we ultimately selected sessions with relatively balanced classes for analysis.

We believe this screening strategy is reasonable because it ensures that the model can truly learn neural activity patterns related to decision-making, rather than simple statistical biases. In the future, we will continue to explore more effective methods for handling imbalanced data, which is also an important direction for our subsequent research.

Regarding the reviewer's question about binning of spike times and the concerns raised by Figure 4b, where accuracy curves for most methods do not improve with increased bin numbers and some even decline, making it difficult to justify 7 bins as optimal:

We appreciate the reviewer's insightful observation about the relationship between bin number and model performance. While increasing the number of bins theoretically provides finer temporal dynamic features, this relationship is not strictly monotonic due to several competing factors:

First, as the number of bins increases, the neural spike data becomes increasingly sparse within each bin, making it more challenging to capture meaningful feature information between spikes. This sparsity can introduce noise and reduce the signal-to-noise ratio in each temporal segment, potentially degrading model performance.

Second, our binning operation equally divides the decision time, which inherently represents a trade-off in temporal dynamic feature representation. Too few bins may obscure important temporal patterns, while too many bins may fragment coherent neural activity patterns.

Figure 4b primarily demonstrates the robustness of our CA-BiLSTM method across different bin configurations rather than identifying an absolute optimal number. The selection of 7 bins represents a balanced compromise based on comprehensive testing across the range of 2-10 segments. This choice balances temporal resolution against data sparsity concerns.

Importantly, we observed during our experiments that different mice exhibit different optimal bin numbers, likely reflecting individual variations in neural processing speed and decision-making dynamics. The 7-bin configuration provides generally good performance across the population while remaining computationally efficient.

The relative stability of performance across different bin numbers actually demonstrates the robustness of our approach, particularly for the CA-BiLSTM model, which maintains higher performance than comparison methods across all bin configurations. This stability suggests that our method effectively captures the most essential temporal dynamics even with moderate temporal resolution.

We greatly appreciate the reviewer's profound insights regarding the interpretation of reaction time analysis results. The reviewer raised a key issue, questioning our simple attribution of the relationship between model prediction results and reaction time to a "speed-accuracy trade-off (SAT)".

The reviewer is entirely correct; our previous explanation indeed contained conceptual confusion. We now recognize that the reaction time analysis in our study compares the differences in mice reaction times between "trials correctly predicted by the model" and "trials incorrectly predicted by the model," rather than the traditional speed-accuracy trade-off in the mice's own correct versus incorrect judgments. This is an important conceptual distinction.

Based on the refined analysis in Figure 7c, we can now present these findings more accurately:

When controlling for stimulus contrast, we observed that across most contrast levels, trials correctly predicted by our model typically had slightly longer mouse reaction times, with an average difference of 11 milliseconds. This pattern was particularly evident at medium contrast levels (such as 0.25), while at extremely low contrast (0.0625) conditions, this difference became minimal.

Regarding stimulus intensity, the data shows that high-contrast stimuli are associated with more trials correctly predicted by the model, with the fewest model prediction errors at the highest contrast (1.0). This distribution aligns with expected behavioral outcomes, as high-contrast stimuli typically enable mice to make more accurate judgments.

We believe these findings suggest that decision processes with longer reaction times may produce more stable, richer neural activity patterns, providing more interpretable information for our decoding model. This observation emphasizes the importance of temporal dynamics of neural activity in extracting decision-relevant information, rather than a simple speed-accuracy trade-off.

We have revised the relevant descriptions in our revised manuscript, removing inappropriate SAT references and clarifying that we are actually analyzing the relationship between model prediction results and mouse reaction times. We thank the reviewer for pointing out this important conceptual distinction, which helps interpret our research findings more accurately.

Comment 2.4

I appreciate the additional details regarding the task structure and the presence of the blocks. I could not find, however, where the analysis of the block structure is incorporated in the manuscript, as the authors claim. Regarding Figure 7 c, since the contrast levels are discrete, a histogram would have been a more appropriate visualization for its distribution.

Response 2.4: Thank you for the comment.

We thank the reviewer for their careful evaluation and for highlighting the need for clarity regarding the incorporation of the block structure analysis in our manuscript. We apologize for any ambiguity in our previous response. To address this concern, we have carefully reviewed the manuscript and ensured that the analysis of the block structure is explicitly incorporated and clearly described.

Specifically, the block structure, which refers to the organization of trials into blocks with distinct stimulus probability conditions (P(Left) = 0.2, 0.5, 0.8), is central to the behavioral and neural decoding analyses presented in Figure 8 (as detailed in the revised manuscript). The block structure is analyzed in the context of its influence on response times and prediction accuracy, as follows:

Behavioral and Neural Decoding Patterns (Fig. 8a,b): We demonstrate how the block structure, defined by the stimulus probability conditions, modulates the speed-accuracy trade-off in mouse behavior and the temporal dynamics of neural decodability. The distinct probability conditions within each block (P(Left) = 0.2, 0.5, 0.8) lead to significant differences in response times, with the most pronounced effects observed at P(Left) = 0.8, as described in the results section.

Model Prediction Accuracy (Fig. 8c): The block structure directly impacts model prediction accuracy, with higher accuracy observed in blocks with extreme probability conditions (P(Left) = 0.2 and 0.8) compared to the balanced condition (P(Left) = 0.5). This is attributed to reduced decision uncertainty in blocks with more predictable stimulus probabilities.

Consistency Across Conditions (Fig. 8d,e): We also analyzed whether the block structure influenced other performance metrics, but no clear trends were observed, potentially due to limited data volume, as noted in the manuscript.

To ensure clarity, we have revised the manuscript and added this content to the Result Analysis section of the main text. In addition, as you suggested, we have changed the scatter plot in Figure 7C to a histogram representation that is more suitable for discrete contrast levels.

Comment 2.5

There isn't still a justification of why the model SVM_RBF is appropriate to perform the analysis in Figure 6. The result should still be introduced more with the character of a validation of the obtained attention weights rather than a result in itself showing evidence for the superiority of the method over others. Probably the same results would have been obtained using one of the other techniques that I'm about to discuss. In fact, since the SVM_RBF doesn't employ the attention mechanism itself, just a selection of the neurons based on the attention weights, appropriate regularization should be able to find a similar selection of neurons if that increases the performance of the model SVM_RBF. Regarding the new analysis in Figure 7 a and b, I'm missing details about what was exactly done in each case (L1-LogReg, L1-MLP, ANOVA, RFE). There are no references or explanations provided whatsoever. Therefore I'm unable to extract much from this result, despite it showing some evidence in favour of superior performance of the CA-BiLSTM selection. Finally, Figure 7d shows some appreciated additional information regarding the distribution of "important neurons" across brain regions. However, the discussion about it is confusing. The highlighted regions DG, LP and PPC aren't among the ones with highest weights, they sit rather on the bottom half of the plot (not DG). The authors don't elaborate much on the implications of this result, which could be relevant for the broad Neuroscience community.

Response 2.5: Thank you for the comment.

We thank the reviewer for their insightful comments regarding the use of the SVM_RBF model in validating neuronal selection and the lack of detailed descriptions for the alternative neuronal selection methods in Figs. 7a and 7b. These valuable suggestions have enabled us to refine the explanation of our methodological choices and the presentation of our results.

Regarding the choice of SVM_RBF as a validation tool, we fully agree with the reviewer that its role should be framed as a validation of attention weights rather than as standalone evidence of our method's superiority over others. We selected SVM_RBF for the following reasons: First, it is a widely validated nonlinear classifier with proven efficacy in neural data classification (Song et al., 2011). Second, unlike our BiLSTM model, SVM_RBF lacks temporal modeling capabilities, providing an independent cross-validation tool to confirm whether selected neurons contain decision-relevant information, rather than being selected due to specific architectural biases of temporal models. Additionally, SVM_RBF's computational efficiency enabled extensive cross-validation experiments across multiple thresholds and selection methods. Finally, in our preliminary experiments, SVM_RBF outperformed other machine learning methods (Figs. 4a, b). To address the reviewer's point that similar results might be achievable with appropriate regularization, we have included BiLSTM as a secondary validation tool (Fig. 7b), further confirming that neurons selected via our attention mechanism perform robustly across diverse model architectures.

For the comparisons in Figs. 7a and 7b with other neuronal selection methods, we acknowledge the lack of detailed descriptions in the original manuscript. Below, we provide detailed explanations of the methods used: L1-regularized logistic regression (L1-LogReg) was implemented using scikit-learn's LogisticRegression with L1 regularization (penalty='l1', C=0.01), which enforces sparsity by driving irrelevant feature weights to zero (Islam et al., 2024). L1-regularized multi-layer perceptron (L1-MLP) was implemented in PyTorch with a single hidden layer (128 units) and L1 penalty ($\lambda = 0.01$) on input layer weights, extending feature selection to nonlinear contexts (Mohammadi, 2021). Analysis of variance (ANOVA)

utilized scikit-learn's f_classif function to compute F-statistics between each neuron and output categories, selecting neurons with the highest F-values, a widely used statistical filtering method in neural signal analysis (Raufi & Longo, 2024). Recursive feature elimination (RFE) was implemented using scikit-learn's RFE with an SVM classifier, iteratively removing the least important features to provide a model-based selection strategy (Dip et al., 2025). All methods were configured to select the same number of neurons as CA-BiLSTM for fair comparison, with selected neuron subsets subsequently used as inputs to SVM_RBF and BiLSTM validation models.

Our results show that neurons selected by the CA-BiLSTM attention mechanism outperformed those selected by other methods across all tested conditions, based on accuracy and AUC metrics. This suggests that our attention mechanism captures more meaningful neural representations relevant to decision-making, rather than merely identifying arbitrary feature subsets that improve specific model performance. Importantly, these findings validate the efficacy of our attention weights rather than directly proving the overall superiority of the CA-BiLSTM approach. We will incorporate these details and relevant references into the revised manuscript, clearly emphasizing the validation role of SVM_RBF analyses to ensure readers fully understand the significance of our methodological choices and comparative results.

Thank you for the reviewer's careful observation and valuable suggestions regarding Figure 7d. Your confusion about the brain region distribution results of "important neurons" is very reasonable, and we would like to provide a detailed explanation of the causes and significance of this phenomenon.

As you observed, although the DG, LP, and PPC regions are widely recognized in existing neuroscience literature as playing important roles in visual decision-making processes, they do not rank at the top in our average attention score rankings. This seemingly contradictory phenomenon actually reflects the influence of data sampling strategies and statistical methods:

First, our data is based on the IBL dataset, which primarily conducts systematic recordings from five core brain regions (CA1, PO, DG, LP, and PPC). These regions are recorded in nearly all experimental mice and typically contain large numbers of neurons. Due to the large sample size, most neurons in these regions have relatively low attention weights, thereby reducing the overall average scores.

In contrast, some non-primary target regions (such as the Anterior Pretectal Nucleus (APN), Suprageniculate Nucleus (SGN), and Ventral Posteromedial Nucleus of the thalamus (VPM)) are recorded in only a subset of mice with fewer neurons. Among these limited samples, a few neurons happen to have very high attention weights, causing the average attention scores of these regions to be significantly elevated.

From a neuroscience perspective, this result has important implications. Despite relatively low average attention scores, the importance of DG, LP, CA1, and PPC regions in decision-making processes is consistent and reliable, which is highly consistent with existing research literature. The "importance" of these regions is reflected in their functional consistency and universality, rather than extremely high weights of individual neurons.

To ensure analytical rigor and reliability of conclusions, we focused our analysis on the five primary target brain regions of the IBL dataset. These regions have sufficient neuronal sampling to support meaningful statistical inference. While those non-primary regions showing high average attention scores have potential research value, they require further

validation in future specifically designed studies due to sample size limitations and possible sampling biases regarding their role in visual decision-making.

Comment 2.6

Detailed comments
Most of these are addressed. I have some additional comments. The caption of Fig 5 still isn't very clear for me. "Trial select" and "select" are strange labels for the y-axis. The relationship between the three variables shown is still not clearly communicated.

Response 2.6: Thank you for the comment.

We thank the reviewer for their continued feedback. Your observation that the titles and labels in Figure 5 remain insufficiently clear is very pertinent. We agree that the labels "Trial select" and "select" are not intuitive, and the relationships among the three variables shown in the figure require more explicit explanation.

We will make the following modifications to Figure 5:

The y-axis label of the upper graph will be changed to "Attention Weight," clearly indicating that these are neuronal attention weight values.

The middle heatmap label will be changed to "Test Trials."

The lower graph label will be changed to "Avg. Weight," with an added explanation that only positive weight values are displayed.

Additionally, we will revise the title and legend of Figure 5 to more clearly explain the relationships among the three subplots: the upper graph shows the overall attention distribution of individual neurons, the middle heatmap details the specific attention weights of each neuron in every trial of the test set, and the lower graph visualizes the average attention weights of neurons across all trials, retaining only positive weight values to more intuitively observe the relative importance of neurons.
