# Supplementary material for: Decoding decision-making behavior from sparse neural spiking activity
Source: PLoS Comput Biol. 2025 Aug 21;21(8):e1013335. doi: 10.1371/journal.pcbi.1013335 (PMC12416849; doi:10.1371/journal.pcbi.1013335)
Supplement: Attachment — Submitted filename: review_response_auresp_3.pdf [file pcbi.1013335.s009.pdf]

Responses to Reviewers' Comments for Manuscript
PCOMPBIOL-D-24-01470R3

Decoding Decision-Making Behavior from Sparse Neural Spiking Activity

Addressed Comments for Publication to

PLOS Computational Biology

by

Yuhang Zhang, Tao Sun, Boyang Zang and Wan Sen

Dear Dr. Daniele Marinazzo,

Please find enclosed the revised version of our previous submission entitled "Decoding Decision-Making Behavior from Sparse Neural Spiking Activity" with manuscript number PCOMPBIOL-D-24-01470R3. We would like to thank you and the reviewers for the valuable comments which help improving the quality of our manuscript. In this revision, we have carefully addressed the reviewers' comments. A summary of main modifications and a detailed point-by-point response to the comments from Reviewer 2 are given below.

Sincerely,

Yuhang Zhang, Tao Sun, Boyang Zang and Wan Sen

Note: To enhance the legibility of this response letter, all the editor's and reviewers' comments are typeset in boxes. Rephrased or added sentences are typeset in color. The respective parts in the manuscript are highlighted to indicate changes.

Response to the Meta Review

Summary Comment 1

Please include the authors' affiliations in the online submission form. Please ensure that the affiliations of the authors listed on the manuscript title page do exactly match with the affiliations provided in the online submission form.

Response S1: Thank you for the comment.

Thank you for bringing this important matter to our attention. We understand the requirement to ensure complete consistency between the author affiliations listed on the manuscript title page and those provided in the online submission form.

Response to Reviewer 1

Comment 1.1

I congratulate the authors for their effort, which have lead to significant improvements in the manuscript. My comments have been largely addressed.
I don't understand the last panel of Fig 7c (bottom right). How is it normalized? Neither the total correct+error sums to 1 in each bin, nor each conditional distribution of corrercts or erros sums to 1 either. Please revise that detail.

Response 1.1: Thank you for the comment.

We sincerely thank the reviewer for this insightful comment and for bringing this important clarification to our attention. We appreciate the reviewer's careful examination of our figure, which has helped us identify an area where our presentation could be clearer.

To clarify the normalization in Figure 7c (bottom right panel), we show the distribution of correct and incorrect predictions across different stimulus contrasts. The normalization was performed by dividing all counts by the single maximum count value (Correct Stimuli = 1) across all stimulus intensities (both correct and incorrect predictions). This normalization method was chosen to facilitate visual comparison of prediction performance patterns across different stimulus intensities while preserving the relative magnitude differences between correct and incorrect predictions.

We have now added this clarification to the figure caption to avoid confusion for future readers. We are grateful for the reviewer's attention to detail, which has helped us improve the clarity of our manuscript.
